# Description of a new species of *Distenia* (Coleoptera, Disteniidae, Disteniini) from Southeastern China, with records and diagnoses of similar species

**DOI:** 10.3897/zookeys.275.4700

**Published:** 2013-03-04

**Authors:** Wen-Xuan Bi, Mei-Ying Lin

**Affiliations:** 1 Key Laboratory of Zoological Systematics and Evolution, Institute of Zoology, Chinese Academy of Sciences, Beichen West Road, Chaoyang Dist., Beijing, 100101, China; 2 Room 401, No. 2, Lane 155, Lianhua South Road, Shanghai, 201100 China

**Keywords:** *Distenia orientalis*, new species, taxonomy, Oriental region, Disteniidae

## Abstract

A new species, *Distenia orientalis* **sp. n.** is described from Southeastern China. It was misidentified as *Distenia gracilis* (Blessig, 1872) but can be separated from the latter by the color of antennae and legs, structure differences on scape, maxillary palp, pronotum, tibiae, punctures on elytra, etc. Three related species are carefully diagnosed and treated.

## Introduction

During research on the fauna of Tianmushan, the first author, Wenxuan Bi, experienced difficulties with identification of *Distenia gracilis* (*sensu* Gressitt 1951; Chen et al. 1959). The fresh material he collected from Tianmushan of Zhejiang Province seems very different from that from Northeastern China and continental Russia. After studying further material from different localities, we conclude that there are three species among the specimens hitherto identified as *Distenia gracilis*: *Distenia gracilis* (Blessig, 1872),*Distenia japonica* Bates, 1873 and *Distenia orientalis* sp. n.

Material studied is deposited in the following institutions and private collections:

**CBWX** Collection of Wenxuan Bi, Shanghai, China

**CCCC** Collection of Chang-chin Chen, Tianjin, China

**CJM** Collection of Ming Jin, Shanghai, China

**CYZZ** Collection of Zhizhou Yu, Shanghai, China

**CZDY** Collection of Deyao Zhou, Shanghai, China

**IZAS** Institute of Zoology, Chinese Academy of Sciences, Beijing, China

**MD** Collection of Mikhail L. Danilevsky, Moscow, Russia

**NHML** The Natural History Museum, London, UK

**SNUC** The Insect Collection of Shanghai Normal University, Shanghai, China

**ZMAS** Museum of Zoology, Academy of Sciences, Saint-Petersburg, Russia

**ZMMU** Zoological Museum of Moscow University, Moscow, Russia

## Results

### Distenia gracilis

(Blessig, 1872)

http://species-id.net/wiki/Distenia_gracilis

**Figures 1–4.**
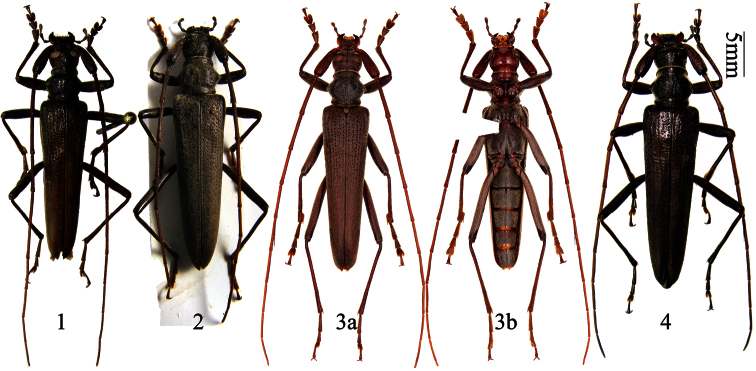
*Distenia gracilis* (Blessig, 1872). **1** male, from Far East Russia **2** female, from Far East Russia **3** male, from Liaoning, China **a** dorsal view **b** ventral view **4** female, from Liaoning, China. Scale 5 mm.

Figs 1 [Fig F2] –15 37–38

Apheles gracilis Blessig, 1872: 168, pl. VIII, fig. 1; Ganglbauer 1887: 131.
Distenia gracilis : Kraatz 1879: 91; Plavilstshikov 1936: 105, 492, fig. 70; Gressitt 1951: 45 [part]; Chen et al. 1959: 32, Pl. III, fig. 16 [part]; Lee 1987: 9, pl. I, fig. 1; Švácha and Danilevsky 1987: 38 [part]; Cherepanov 1990: 68; Hua 2002: 189 [part]; Hua et al. 2009: 448 [part]; Lin et al. 2010: 120 [part]; Danilevsky 2012: 902.

#### Host plant.

*Alnus* sp. (BETULACEAE), *Chosenia* sp. (SALICACEAE) (Danilevsky 2012).

#### Remarks.

This species was first recorded from Northeastern China (Manchuria) by Plavilstshikov (1936). Gressitt (1951) cited this information and added Zhejiang (Tianmushan) as a new locality, which was the first misidentification. Then, Chen et al. (1959) followed Gressitt (1951) and made a drawing based on specimens from Tianmushan, which misled subsequent Chinese longicornists to misidentify *Distenia orientalis* sp. n. as *Distenia gracilis*. Therefore, the record from Zhejiang and Jiangxi is incorrect, as it was based on misidentification of *Distenia orientalis* sp. n. The records from Hubei and Anhui are doubtful and may also be based on misidentification of *Distenia orientalis* sp. n. (or another species) but we did not have specimens available from these two provinces. Chou (2004) didn’t include *Distenia gracilis* in his book on Taiwanese fauna. Records of *Distenia gracilis* from Japan were based on misidentification of *Distenia japonica*.

We did not have specimens from Korea for study. We consider the record by Ganglbauer (1887) and Lee (1987) correct based on the pictures by Lee (1987).

The holotype of *Apheles gracilis* Blessig, 1872 is a male from Russia, Sibérie (Amurland), collected by P. Wulffius. It was supposed to be deposited in ZMAS. We could not reach the curators in ZMAS. According to personal communication by Mikhail Danilevsky, he could not find the type in the collection of ZMAS.

#### Distribution.

North China (Heilongjiang, Jilin, Liaoning), Korea (including South Korea and North Korea), Russia (Far East).

#### Specimens examined.

**China, Liaoning:** 2 females, Benxi, Guanmenshan, 2011.VIII.21, coll. Xinlei Huang (IZAS); 1 male 1 female, Dandong, Saima, Wendong, 2006.IX.1, 3, coll. Haicheng Shan (IZAS, ex CCCC); 2 males, Dandong, Saima, Pushihe, 2008.VII.30, coll. Haicheng Shan (CBWX).

**Russia, Far East:** 1 male, Arsenyev env., 44°7'27"N, 133°20'00"E, 2007.VII.21, coll. S. Ivanov (MD); 1 male, Primorie Reg., Chernigovka distr., Merkushevka Env., 44°22'2.52"N, 132°48'0.42"E, 2011.VII.28–30, coll. S. Ivanov (MD).

**Figures 5–11. F2:**
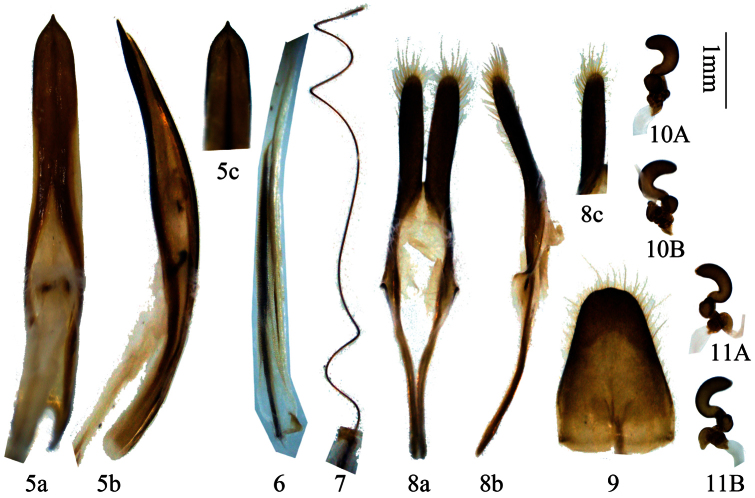
Genitalia of *Distenia gracilis* (Blessig, 1872). **5–9** male, from Far East Russia **5** median lobe **6** rods of endophallus **7** hair-like thin rod of ejaculatory duct **8** tegmen **a** ventral view **b** lateral view. **c** dorsal view **9** tergite VIII in dorsal view **10–11** female, spermathecal capsule, both from Liaoning, China. **A–B** from different sides. Scale 1 mm.

**Figures 12–15.**
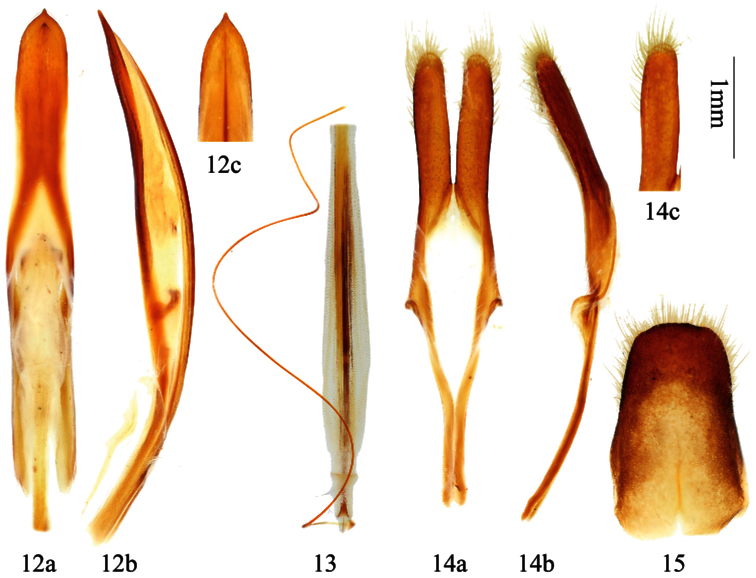
Genitalia of *Distenia gracilis* (Blessig, 1872), male, from Liaoning, China **12** median lobe **13** rods of endophallus, including hair-like thin rod of ejaculatory duct **14** tegmen **a** ventral view **b** lateral view **c** dorsal view **15** tergite VIII in dorsal view. Scale 1 mm.

### Distenia japonica

Bates, 1873

http://species-id.net/wiki/Distenia_japonica

Figs 16 –24 39–40

Distenia japonica Bates, 1873: 155.
Distenia gracilis : Kraatz 1879: 91; Švácha and Danilevsky 1987: 38 [part]; Lin et al. 2010: 120 [part].
Distenia gracilis gracilis : Ohbayashi and Niisato 2007: 335, pl. 1, figs 1 (male) & 2 (female) [Fauna].
Distenia japonica : Danilevsky 2012: 902.

#### Host plant.

It is polyphagous with the following host plants recorded under *Distenia gracilis* (confused with *Distenia japonica*): *Acer* sp. (ACERACEAE), *Abies sachalinensis* Masters (PINACEAE), *Alnus* sp. (BETULACEAE), *Betula* sp. (BETULACEAE), *Chosenia* sp. (SALICACEAE), *Picea* sp. (PINACEAE), *Pinus* sp. (PINACEAE), *Quercus* sp. (FAGACEAE), *Salix* sp. (SALICACEAE), *Ulmus* sp. (ULMACEAE).

#### Diagnosis.

According to Danilevsky (2012), *Distenia gracilis* Blessig, 1872 (mainland and Sakhalin) and *Distenia japonica* Bates, 1873 (islands) are different vicariant species, very easily distinguished by narrow scapus in *Distenia japonica*. Further differences are shown in Table 1.

#### Remarks.

This species was first described by Bates (1873) based on syntypes from Japan, Honshu (Hyogo Prefecture), Maiyasan, collected by George Lewis. Kraatz (1879) synonymized it with *Distenia gracilis*, which was widely followed by subsequent authors until Danilevsky (2012) resurrected it.

Švácha and Danilevsky (1987) pointed out the habit differences between the mailand population and island population, and suspected “it is possible that we are facing two separate taxa”. “However, reliable larval morphological differences have not been found.” (Švácha and Danilevsky 1987). According to Danilevsky (2012), *Distenia gracilis* (mainland and Sakhalin) develops underground on healthy roots of living *Chosenia* (personal observation in Kedrovaya Pad) and on *Alnus*, but *Distenia japonica* lives under the old dead bark of many different trees (personal observation on Kunashir), often together with *Eutetrapha*. Therefore, the host plants recorded under *Distenia gracilis* could actually be host plants of *Distenia japonica*.

#### Distribution.

Japan, Russia (Far East, Islands).

#### Specimens examined.

**Japan:** 1 male, syntype, Japan (NHML, ex collection G. Lewis, examined through pictures); 1 male, Japan, Iwate Prefecture, Niisato-mura, Genbeidaira, 1982.VII.31, coll. N. Ohbayashi (CBWX); 1 female, Japon, Iwate Prefecture, Niisato-mura, Genbeidaira, 1982.VII.31, coll. N. Ohbayashi (CBWX); 1 male 1 female, Kyoto, Kibone, 1932.VII.1, coll. S. Yie (IZAS); 1 female, Tokushima, Mt. Tsurugi, 1971.VII.11, coll. H. Toshima (IZAS); 1 female, Tottori Pref., Mt. Hokki-Daisan, 1958.VII.22, coll. H. Toshima (IZAS).

**Table 1.** Differences of *Distenia gracilis*, *Distenia japonica* and *Distenia orientalis* sp. n.

Species / Character	*Distenia gracilis*	*Distenia japonica*	*Distenia orientalis* sp. n.
Antennal segment extending beyond tip of elytra	in male 8^th^, in female 9^th^	in male 8^th^, in female 9^th^	in male 7^th^, in female 8^th^
Color of antennae and legs	uniformly black-brown	Uniformly brown	Mostly black-brown, with several orange-red rings
Scape in male	With basal grooves, punctures coarser	With basal grooves, punctures finer	Without basal grooves, with rugose punctures
Scape length / maximum width	ca.3.0 in male, ca. 2.8 in female	ca.3.5 in male, ca. 3.0 in female	ca.3.1 in male, ca. 3.4 in female
Last segment of maxillary palp	Stouter, length / maximum width < 2.5 in male, < 2.6 in female (Figs 37a, 38a)	Stoutest, length / maximum width < 2.1 in male, < 2.4 in female (Figs 39a, 40a)	Slender, length / maximum width > 2.5 in male, > 3.0 in female (Figs 41a, 42a)
Pronotum	Without transverse rugae, swelling indistinct (Figs 37c, 38c)	Without transverse rugae, swelling more distinct (Figs 39c, 40c)	With some transverse rugae (Figs 41c, 42c)
Mosotibiae of male	Apical protruding lobe very distinct (Fig. 37f )	Apical protruding lobe distinct (Fig. 39f)	Without apical protruding lobe (Fig. 41f)
Punctures on elytra	With distinct longitudinal rows, the row near suture not very dense (Figs 37d, 38d)	With distinct longitudinal rows, the row near suture very dense (Figs 39d, 40d)	Longitudinal rows indistinct, the row near suture very sparse (Figs 41d, 42d)
Sternite VII (ventrite V)	Figs 37e, 38e	Figs 39e, 40e	Figs 41e, 42e
Median lobe	Figs 5, 12	Figs 19	Figs 29
Spermathecal capsule	Figs 10–11	Figs 24	Figs 34–36

**Figures 16–18.**
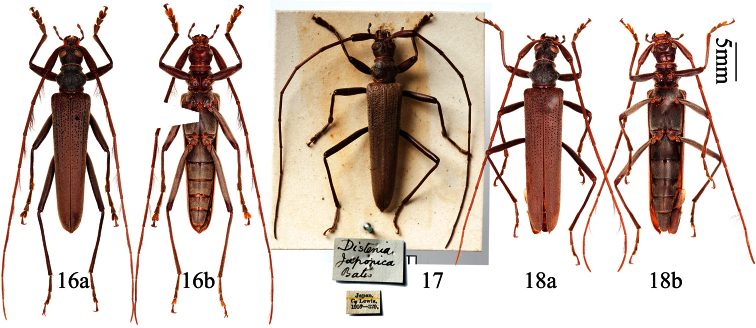
*Distenia japonica* Bates, 1873. **16** male, from Iwate, Japan **17** syntype, male, from Hyogo, Japan **18** female, from Iwate, Japan **a** dorsal view **b** ventral view. Scale 5 mm.

**Figures 19–24.**
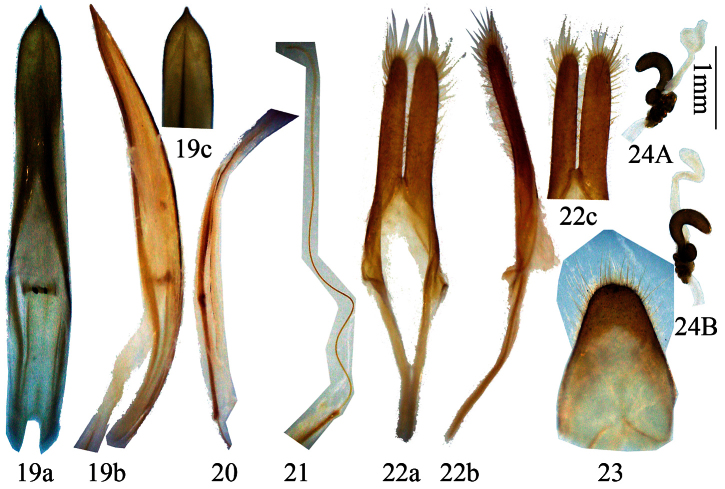
Genitalia of *Distenia japonica* Bates, 1873. **19–23** male, from Kyoto, Japan **19** median lobe **20** rods of endophallus **21** hair-like thin rod of ejaculatory duct **22** tegmen. **a** ventral view **b** lateral view **c** dorsal view **23** tergite VIII in dorsal view **24** female, spermathecal capsule, from Kyoto, Japan. **A–B** from different sides. Scale 1 mm.

### Distenia japonica yakushimana

Yokoyama, 1966

http://species-id.net/wiki/Distenia_japonica_yakushimana

Distenia gracilis yakushimana Yokoyama, 1966: 54, pl. 6, fig. 1.
Distenia gracilis yakushimana : Ohbayashi and Niisato 2007: 336, pl. 1, fig. 3 (male) [Fauna].
Distenia japonica yakushimana : Danilevsky 2012: 902.

#### Diagnosis.

According to Yokoyama (1966): “This subspecies differs from the typical species (*Distenia japonica*), in having the following points: body smaller and more blackish, sparsely covered with shorter brownish yellow pubescence, which is sparser on head and prothorax. Clypeus longer, vertex less punctured. Prothorax weakly irregularly wrinkled, lateral tubercles less developed, not acute at apex. Terminal joint of maxillary palpus rounded at apex (instead of truncate).”

#### Remarks.

This subspecies was described based on the female holotype from Japan, Ryukyu island, Mt. Miyanouradake (alt. 1200 m), collected by Hajime Yokoyama on August 3, 1962. It is deposited in Osaka Museum of Natural History. We did not examine the holotype or other specimens but followed Ohbayashi and Niisato (2007) and Danilevsky (2012) in treating this form as a subspecies.

#### Distribution.

Japan (Yaku-shima).

### Distenia orientalis sp. n.

urn:lsid:zoobank.org:act:14814F4C-97D8-4C2C-9125-7AA5DEDACFA6

http://species-id.net/wiki/Distenia_orientalis

Figs 25 –36 41–42

Distenia gracilis : Gressitt 1951: 45 [part]; Chen et al. 1959: 32, Pl. III, fig. 16; Hua 2002: 189 [part]; Hua et al. 2009: 448 [part]; Lin et al. 2010: 120 [part].

#### Description.

Male: body length 18.7–25.5 mm, width at humeri 4.0–6.0 mm. Female: body length 22.0–26.6 mm, width at humeri 5.0–6.5 mm. Body uniformly black-brown, with rusty tinge (especially in male), except bases of tibiae (about 1/3 to 1/2), tips of antennal segments IV-XI (increasing from IV to XI), and extreme tips of last segments of maxillary and labial palps which are reddish-brown, and ventral side of tarsi and base of mandible being brown.

Body elongate, slender. Head with dense rugose punctures, with mouthparts turned forward and somewhat downward. Last segment of maxillary palp expanded and obliquely truncate apically. Frons between eyes with narrow interrupted longitudinal suture. Antennae long; scape very thick in male and more slender in female, without a groove on basal half, in male with coarse rugose punctures (Fig. 41a), in female not rugose but with finer punctures (Fig. 42a); scape not reaching midlength of pronotum in either sex; pedicel very small; subsequent segments slender; in male 7^th^, in female 8^th^ segment extends beyond tip of elytra; antennal segments with recumbent long hairs beneath. The relative length of antennal segments, male: 10.6:1:12.9:13.2:13.1:12.5:11.9:11.1:9.7:8.7:8.8 (variable in narrow range); female: 9.9:1:10.2:10.3:10.3:10.1:9.5:8.5:7.4:6.5:6.3 (variable in narrow range).

Pronotum broadest in middle, with acute conical lateral spines, near posterior and anterior margins with slight transverse constriction, with rugae on disc, and with dense minute punctures and dense gray pubescence. Scutellum not longer than width at base, apically rounded, with yellowish pubescence.

Elytra narrow, taper uniformly toward apex, length 3.0–3.4 times the total width at humeri, and anterior half with deep punctures forming several indistinct longitudinal rows. Abdominal ventrite V in female (Figs 26b, 34d) elongate, gently rounded posteriorly; in male (Figs 28b, 33d) distinctly emarginate, with minute tender closely recumbent hairs. Legs long and slender, mesotibiae (of both male and female) without apical protruding lobe.

**Male terminalia** (Figs 29–33)**:** Tegmen (Fig. 32) approximately 5.0 mm in length; lateral lobes slender, length about 5 times the width, ventral side and apex with short setae; median lobe plus median struts (Fig. 29) slightly curved, longer than tegmen; the median struts less than 1/8 of the whole median lobe in length; apex of ventral plate bluntly pointed; internal sac bearing a basal armature (Fig. 29b) and two median rods of endophallus (Figs 30, 31), of which the strongly sclerotized one (coming from the gonopore) connected to a very long (much longer than the median rods) hair-like rod (inside ejaculatory duct, Fig. 30). Tergite VIII (Fig. 33) longer than broad, narrowed apically from middle, with rounded apex, apical half bearing short dorsal setae.

**Female terminalia** (Figs 34–36)**:** Paraproct moderate in size, its baculi thick and long, straight and not bifurcate at base; valvifer indistinct; coxite with rough surface, each baculum very thick at base and narrowed towards apex; coxite lobes sclerotized at each inner part, with tactile hairs; stylus articulated to the tip of each coxite lobe (slightly laterally), sclerotized except for apex and bearing tactile hairs; dorsal baculi sinuate and longer than paraproct baculi; proctiger baculi long and almost straight. Spermathecal capsule (Figs 34–36) large, heavily sclerotized and of very intricate structure, its apical part narrow, strongly bent at middle and basally with a protrusion (in shape of a question mark “?”), basal part irregularly twisted and with rather broad protrusion to which attaches the spermathecal gland at the middle part. Tignum much shorter than half of abdomen. In one measured specimen, tignum was 4.4 mm for an adult with 12.0 mm abdomen length in ventral view.

#### Diagnosis.

The differences of the three species are shown in Table 1.

#### Etymology.

The name of the new species refers to its distribution in southeast China, instead of northeast China (which is the distribution of *Distenia gracilis*).

#### Remarks.

This species has been misidentified as *Distenia gracilis* sinceGressitt (1951).

It is the 29^th^ recorded species for the Chinese Disteniidae fauna (Lin et al. 2010; Lin and Murzin 2012).

One female from Mt. Wutaishan of Shanxi Province shows a strange dot on the distributional map. We believe that the distribution region will be extended after further survey.

#### Distribution.

China: Zhejiang Prov., Fujian Prov., Guangdong Prov., Jiangxi Prov., Shanxi Prov.

#### Specimens examined.

**Holotype**, male, Zhejiang, Xitianmushan, alt. 1200 m, 2008.VII.2, coll. Hao Huang (SNUC, ex CBWX). **Paratypes: China, Zhejiang:** 1 male, Xitianmushan, alt. 1300 m, 2009.IV.19 (larva), 2009.V.14 (adult), coll. Wenxuan Bi (CBWX); 1 male, Xitianmushan, alt. 1100 m, 2008.III.1 (larva), 2008.V.27 (adult), coll. Wenxuan Bi (CBWX); 1 female, Tianmushan nature reserve, alt. 1100 m, 2008.VII.30, coll. Yongxiang Wu (CJM); 1 female, China, Chekiang, Tien-mu-shan, 1937.VI.30, coll. E. Surnson (ZMMU); 1 female, Xitianmushan, alt. 1000m, 2012.VII.11, coll. Deyao Zhou (CZDY); 1 female, Tienmushan, 1937.VIII.3 (IZAS, IOZ(E)1859289); 2 males, same data (IZAS, IOZ(E)1859290-91); 2 males, same data but 1937.VIII.4 (IZAS, IOZ(E)1859292-93); 1 male, same data but 1937.VII.21 (IZAS, IOZ(E)1859288); 1 female, Longquan, Fengyangshan, Lu'ao village, alt. 1100 m, 2008.VII.31, coll. Wenxuan Bi (CBWX); Qingyuan county, Baishanzu nature reserve, alt. 1000 m, 2009.VII.25-VIII.5, coll. Zhizhou Yu (CYZZ). **China, Fujian:** 1 male, Chong’an, Sangang, 1979.VIII.14 (IZAS, IOZ(E)1859287); 1 male, Fujian, Wuyishan nature reserve, 2009.VII.10–15. coll. Ming Jin (CJM). **China, Jiangxi:** 1 female, Wuyishan nature reserve, Yejiachang station, alt. 900 m, 2004.VIII.2 (CCCC). **China, Guangdong:** 1 female, Ruyuan county, Nanling nature reserve, 2008-2009, coll. Lei Gao (CCCC).

#### Additional specimen examined.

**China, Shanxi:** 1 female, Wutaishan, alt. 2000 m, 1996.VII.17, coll. Wenzhu Li (IZAS, IOZ(E)1859062).

**Figures 25–28.**
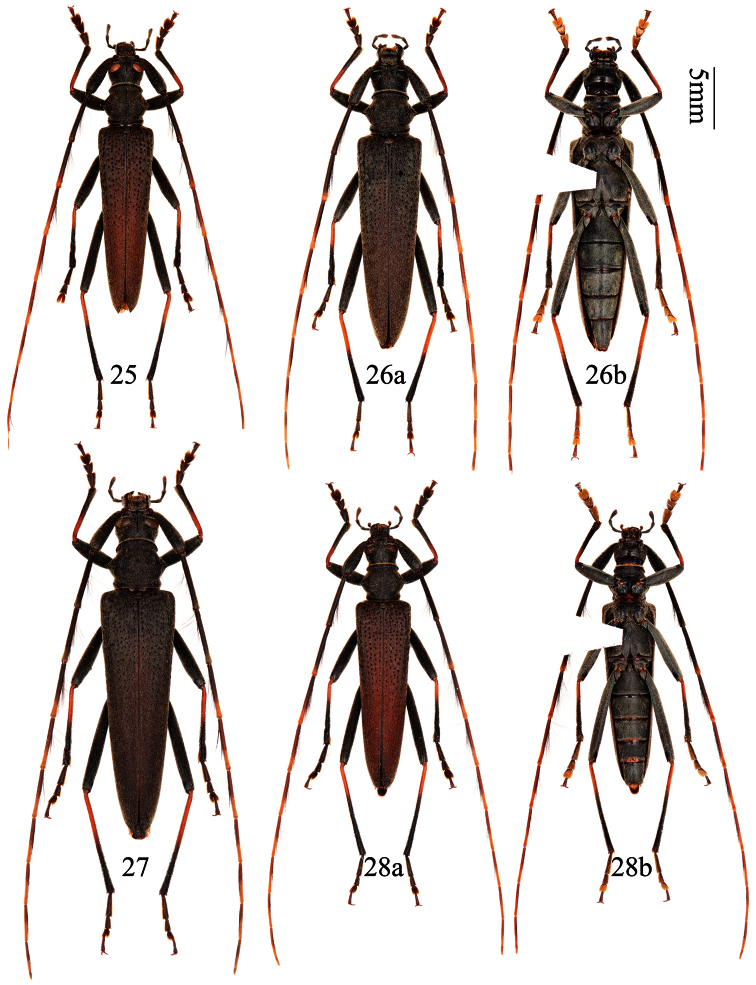
*Distenia orientalis* sp. n. **25** holotype, male, from Xitianmushan, Zhejiang, China **26** paratype, female, from Tianmushan, Zhejiang, China **27** paratype, female, from Fengyangshan, Zhejiang, China **28** paratype, male, from Wuyishan, Fujian, China **a** dorsal view **b** ventral view. Scale 5 mm.

**Figures 29–36.**
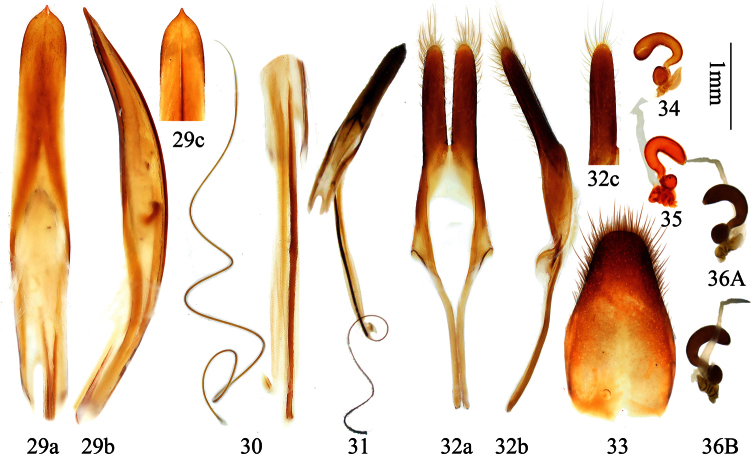
Genitalia of *Distenia orientalis* sp. n. **29–33** male, from Xitianmushan, Zhejiang, China **29** median lobe **30** rods of endophallus and hair-like thin rod of ejaculatory duct **31** whole median lobe, showing the position of rods of endophallus, not to scale **32** tegmen **a** ventral view **b** lateral view **c** dorsal view **33** tergite VIII in dorsal view **34–36** female, spermathecal capsule **35** from Fengyangshan, Zhejiang, China **34** & **36** from Tianmushan, Zhejiang, China. **A** & **B** from different sides. Scale 1 mm.

**Figures 37–42.**
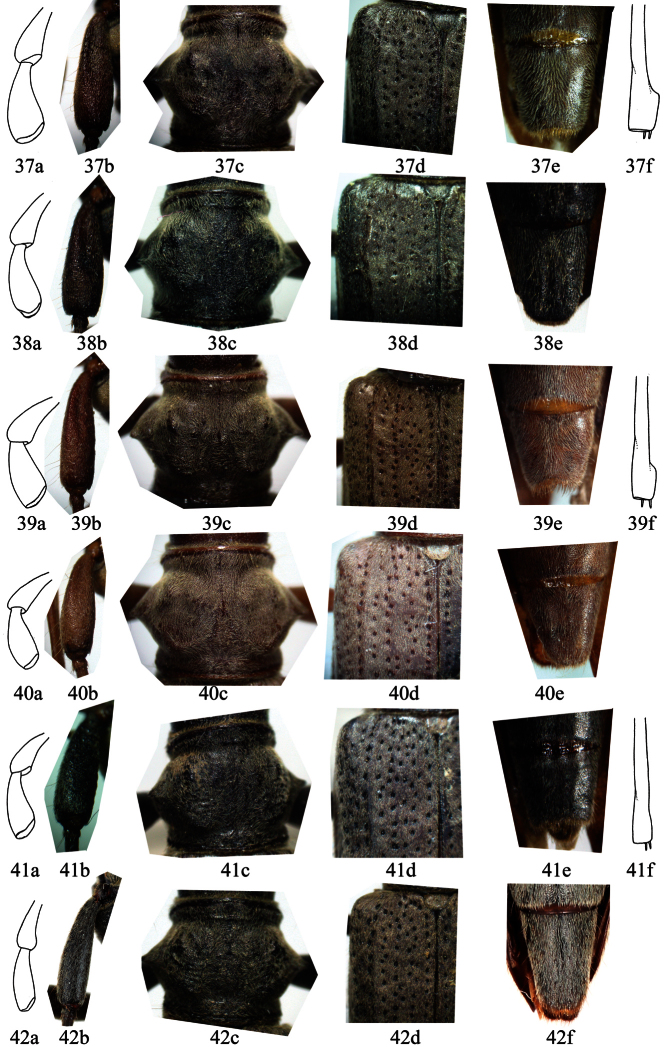
Six important characters of *Distenia* spp. not to scale. **37–38** *Distenia gracilis*. **37** male from Far East, Russia **38** female from Liaoning, China **39–40** *Distenia japonica* **39** male from Kyoto, Japan **40** female from Kyoto, Japan **41–42** *Distenia orientalis* sp. n. **41** male from Tianmushan, China **42** female from Tianmushan, China **a** last segment of maxillary palp, showing the tip and the ration of length to width **b** scape **c** pronotum **d** basal part of elytron **e** ventrite V **f** mesotibia of male, showing the apical protruding lobe.

## Supplementary Material

XML Treatment for Distenia gracilis

XML Treatment for Distenia japonica

XML Treatment for Distenia japonica yakushimana

XML Treatment for Distenia orientalis
